# Crotaline Fab antivenom appears to be effective in cases of severe North American pit viper envenomation: An integrative review

**DOI:** 10.1186/1471-227X-9-13

**Published:** 2009-06-22

**Authors:** Eric J Lavonas, Tammi H Schaeffer, Jamie Kokko, Sara L Mlynarchek, Gregory M Bogdan

**Affiliations:** 1 Rocky Mountain Poison & Drug Center, Denver Health Hospital Authority, 777 Bannock St, MC 0180, Denver, CO 80204, USA

## Abstract

**Background:**

In 2000, the United States Food and Drug Administration approved Crotalidae Polyvalent Immune Fab (Ovine) (hereafter, FabAV), "for the management of patients with minimal to moderate North American Crotalid envenomation." Because whole-IgG pit viper antivenom is no longer available in the United States, FabAV is currently the only specific treatment option available to United States clinicians treating snakebite victims of any severity. No clinical trial data are available concerning the effectiveness of FabAV for treatment of severe snakebite, but several published articles describe its use in this setting.

**Methods:**

We performed a comprehensive review of the English-language medical literature to identify all publications (1996 to July, 2008) containing data about the administration of FabAV. Two trained reviewers separately extracted case-level data concerning the administration of FabAV to patients with severe envenomation by North American crotaline snakes to a standardized form. Descriptive statistics were used. In addition, we hand-searched the US National Poison Data System reports for the years 2000–2006 to identify and describe any reports of death that occurred after FabAV administration.

**Results:**

The literature review found 147 unique publications regarding FabAV. Twenty-four evaluable cases of severe human envenomation treated with FabAV were identified in 19 publications. Seven cases were described in five cohort studies, and 17 cases were described in 14 single patient case reports or non-cohort case series. Sixty-five specific severe venom effects were reported in these 24 patients, of which 50 effects (77%) improved or resolved after FabAV therapy. Initial control of all severe venom effects was achieved in 12 patients (50%). The rate at which initial control was achieved was significantly higher among patients reported in the cohort series than in the case series and non-cohort reports (100% vs. 29%, P = 0.005). The median dose of FabAV used to obtain initial control was 6 vials (range: 4 – 18 vials). Nine patients had severe venom effects that persisted despite FabAV therapy. Recurrent and/or delayed-onset severe defibrination syndrome occurred in 12 patients, most of whom did not receive recommended maintenance FabAV dosing. No patient developed systemic bleeding.

**Conclusion:**

In this structured literature review, FabAV appears to be effective in the management of severe crotaline snake envenomation. Incomplete response to therapy, recurrence of venom effects, and delayed-onset venom effects were reported in case reports, but not reported in cohort studies.

## Background

Each year, envenomation by pit viper snakes (Family *Viperidae*, subfamily *Crotalinae*, genera *Crotalus*, *Agkistrodon*, and *Sistrurus*) causes at least 2,700 people to seek hospital treatment in the United States. About half of these patients receive antivenom[1]. In October 2000, the United States Food and Drug Administration (US FDA) approved a Fab antivenom product for crotaline snakebite. Compared with equine-derived whole-IgG antivenom, Crotalidae Polyvalent Immune Fab (Ovine) (CroFab™, Protherics, Nashville, TN; hereafter, FabAV) is thought to convey a reduced risk of acute and delayed-type hypersensitivity reactions[2].

The US FDA approved FabAV based on two clinical trials, both of which excluded patients with severe envenomation.[3-5] The reason for this exclusion was equipoise: at the time the trials were conducted (1993–96), treating life-threatening venom effects with investigational antivenom *in lieu *of a proven standard therapy was considered unethical. As a result of the trial design, the US FDA approved FabAV, "for the management of patients with minimal or moderate North American crotalid envenomation"[3].

Wyeth Pharmaceuticals announced in 2001 that it would cease production of equine antivenom[6]. It appears that the last lot of equine antivenom expired in April, 2007, and no other antivenom has been approved for treating crotaline snakebite[7]. Therefore, at the present time, there is no approved antivenom therapy for severe crotaline snakebite available in the United States.

Available data suggest that FabAV is being widely used to treat severe envenomations. The 2006 report of the American Association of Poison Control Centers' National Poison Data System (NPDS) lists 2,768 victims of crotaline snake bite treated in a health care facility, of whom five died, 1,528 had moderate to severe toxicity, and 1,239 had minimal or no clinical effects[1]. FabAV was administered to 1,359 patients during this period. In addition, several cohort studies and many case reports have described the use of FabAV to treat snake bite victims of all severities.

We analyzed the English language medical literature to characterize the reported response to FabAV therapy of patients suffering severe crotaline envenomation.

## Methods

We searched PubMed, Ovid MEDLINE, and EMBASE to identify all published articles containing primary data about North American crotalid envenomations treated with FabAV. All searches were performed on July 28, 2008, using the search strategy listed in Table 1. All article types were considered, including prospective clinical trials, cohort and non-cohort case series, single case reports, review articles, editorials, commentaries, published abstracts, and letters-to-the-editor. Citation lists from the three databases were imported into a ProCite database (ProCite^® ^version 5.0 for Windows, ISI ResearchSoft, Philadelphia, PA, USA), and duplicate articles were removed. We then performed an internal search to deselect articles outside the scope of the search. The terms searched were rat(s), mouse, mice, rabbit(s), cellular, *in vivo*, and *in vitro*.

**Table 1:** Search terms

**Database**	**PubMed and Ovid Medline**	**EMBASE**
**Search Terms**	MeSH headings: • Crotalid Venoms ◦ /po (poisoning), ◦ /to (toxicity) • Snake Venoms ◦ /po (poisoning), ◦ /to (toxicity) • Snake Bites ◦ /dt (drug therapy) ◦ /th (therapy) • Viperidae • Agkistrodon • Crotalus	'Crotalid venoms' AND (intoxication OR toxicity) 'Snake venoms' AND (intoxication OR toxicity) 'Snake bites' AND ('drug therapy' OR therapy) Viperidae Agkistrodon
	Keywords: • CroFab • Crotaline immune Fab FabAV	Crotalus Crotaline immune Fab FabAV

All searches were limited to English language, human, and dates of January 1, 1999 – July 28, 2008, inclusive.

A single medical toxicologist (EJL) then reviewed each title and abstract. Articles judged to have no likelihood of containing data about FabAV administration for North American crotalid envenomations (e.g. reports that clearly referred only to the treatment of African, Asian, or Australian snakebite) were excluded from further review.

Two supplemental sources complimented the computerized literature search. Our article list was cross-referenced with the topic-specific article files of Protherics, Inc, the manufacturer of FabAV. The authors also searched the bibliographies of all included articles, major textbooks in emergency medicine and medical toxicology, and our institutional article files for additional primary source materials. These supplemental searches yielded some publications prior to 1999.

Two board-certified medical toxicologists with experience in the management of snakebite (EJL, THS) independently reviewed the full-text version of each article, identified all cases meeting the *a priori *definition of severe envenomation (see below), and extracted case-level data to a standardized form. This form consisted of two pages for data concerning hypersensitivity reactions (collected for a separate manuscript), one page for summary data about any severe envenomation cases, and one additional page for each severely envenomated patient reported. Training on the abstraction step consisted of one hour of instruction, followed by focused review of eleven practice abstractions. The reviewers were not blinded to the intent of the study. Data were entered into a Microsoft Access database (Microsoft Access 2003^®^, Microsoft Corporation, Redmond, WA, 2003) and double verified to ensure accuracy of data entry. Disagreement between the abstractors was identified and resolved by consensus.

Included cases were reviewed to identify duplicate publication; where necessary, study authors were contacted to determine if cases reported in different publications were one and the same.

Data were analyzed using SAS (v. 9.1, SAS Institute, Cary, NC). Descriptive statistics were used; confidence intervals were not presented due to small sample sizes.

The definition of "severe" envenomation was that used in the US FDA-approved prescribing information for FabAV (Table 2). This standard definition contains several areas of ambiguity, which were resolved *a priori *as follows:

**Table 2:** Definitions of Envenomation Severity from the FabAV Prescribing Information[3]

**Envenomation Category**	**Definition**
Minimal	Swelling, pain, and ecchymosis limited to the immediate bite site; Systemic signs and symptoms absent; Coagulation parameters normal with no clinical evidence of bleeding.

Moderate	Swelling, pain, and ecchymosis involving less than a full extremity or, if bite was sustained on the trunk, head or neck, extending less than 50 cm; Systemic signs and symptoms may be present but not life threatening, including but not limited to nausea, vomiting, oral paresthesia or unusual tastes, mild hypotension (systolic blood pressure >90 mmHg), mild tachycardia (heart rate <150), and tachypnea; Coagulation parameters may be abnormal, but no clinical evidence of bleeding present. Minor hematuria, gum bleeding and nosebleeds are allowed if they are not considered severe in the investigator's judgment.

Severe	Swelling, pain, and ecchymosis involving more than an entire extremity or threatening the airway; Systemic signs and symptoms are markedly abnormal, including severe alteration of mental status, severe hypotension, severe tachycardia, tachypnea, or respiratory insufficiency; Coagulation parameters are abnormal, with serious bleeding or severe threat of bleeding.

The standard definition of local tissue effects classifies, "swelling, pain, or ecchymosis involving less than a full extremity," as "moderate," and, "swelling, pain or ecchymosis involving more than an entire extremity," as "severe." We included cases involving swelling, pain, or ecchymosis involving exactly an entire extremity in the "severe" group.

The standard definition of neurotoxicity classifies, "oral paresthesias or unusual tastes," as "moderate," but has no criteria for severe neurotoxicity. We considered severe muscle weakness, difficulty speaking or swallowing, and fasciculations remote from the bite site (sometimes a sign of impending paralysis) to be signs of severe neurotoxicity.

The phrase, "coagulation parameters are abnormal, with serious bleeding or severe threat of bleeding," in the standard definition required more precise definition. Using published criteria, we considered "severe threat of bleeding" to be present if the platelet count was less than 50,000 cells/mm^3^, if the fibrinogen was less than 50 mg/dl (1.5 micromol/L), or if the international normalized ratio (INR) or protime ratio were > 5.0[8] If protime was reported without INR or normal range data, a protime > 50 seconds was considered to represent severe threat of bleeding.

The standard definition does not specify a time point at which severity is assessed. In order to mirror clinical practice, we graded the severity of envenomation based on the initial presentation, i.e. the patient's clinical condition during the first six hours after presentation for care. Cases that were of minimal or moderate severity on initial presentation, but that developed one or more features of severe envenomation many hours or days later, were counted as "minimal" or "moderate" based on the severity of the initial presentation.

In some cases, authors reported clinical manifestations such as, "severe tachycardia," or, "hypotension," without reporting numeric values. We counted these cases as, "severe," if the author of the report described them as, "severe."

The Snakebite Severity Score (SSS) is a validated tool for assessing crotaline snake envenomation[9]. A SSS ≥ 7 is generally considered to represent severe envenomation. Although clinicians' global assessment of severity was used to determine eligibility in the FabAV premarketing trials, serial calculations of SSS were made in all patients as the primary efficacy outcome of the premarketing trials and at least one other report[4,5,10] Although the SSS is a composite measure of severity, rather than a unique venom effect, serial calculations of SSS were the only case-level data presented about response to treatment in some reports[4,5,10]. Therefore, when data were available, we calculated the SSS for each case reported and included those with a SSS ≥ 7 in the "severe" group.

In this report, we defined "initial control" of a specific venom effect, *a priori*, as cessation of progression of local tissue effects (pain and swelling), and as complete resolution of systemic effects (hypotension, neurotoxicity, and medically important bleeding). In accordance with previous research, initial control of coagulopathy was defined as definite improvement in coagulopathy or platelet count tests, as appropriate, combined with the absence of systemic bleeding[5,8,11-13]. "Initial control of the envenomation syndrome" was defined *a priori *as simultaneous initial control of all specific venom effects experienced by that particular patient.

After initial control of the envenomation syndrome is achieved, the manufacturer of FabAV recommends administration of 2-vial doses of FabAV, given at 6, 12, and 18 hours ("maintenance therapy"), in order to prevent recurrent or delayed onset venom effects[3,5]. We scored a patient as having received maintenance therapy if the patient received at least three doses of FabAV, each consisting of two or more vials, within a 24-hour period after initial control was reached.

Annual reports of the American Association of Poison Control Centers' NPDS, and its predecessor, the Toxic Exposure Surveillance System (TESS), contain robust statistical data about poisonings in general, and brief (< 100 word) case abstracts about fatal poison exposures. Because they do not focus on snakebites, NPDS/TESS reports were expected neither to be identified by the search strategy, nor to contain sufficient detail to be included in the main analysis. Nonetheless, they remain a relevant, important source of peer-reviewed data. Therefore, we hand-searched the 2000–2006 annual reports for any reported fatalities that occurred after FabAV administration[1,14-19].

## Results

### Article and subject identification

**Figure 1:**
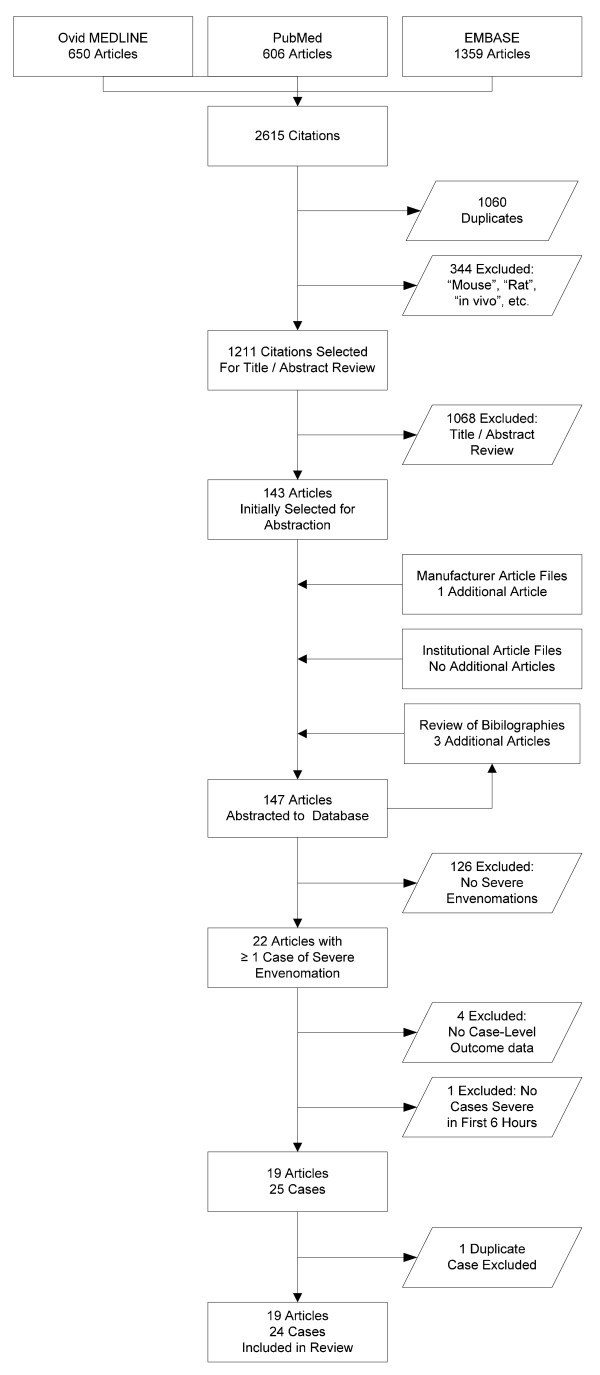
**Article identification and selection process**. This information is also presented as an attached file.

The search strategy identified 147 unique publications regarding FabAV. The article selection process is depicted in Figure 1. A total of 22 articles contained at least one case of severe envenomation by a North American crotaline snake treated with FabAV. These articles describe 30 apparently-unique cases of severe envenomation. From this group, five cases did not contain sufficient data about the clinical course after FabAV administration to judge whether the manifestations of severe envenomation responded to therapy, and one case was determined to be included in two different series; these cases were therefore excluded[10,20-24]. The remaining 24 cases from 19 published reports are presented in Table 3[4,10,13,20,22,25-38].

**Table 3:** Published cases of severe envenomation treated with FabAV

**Publication**	**Patient Identifying Information**	**Severe venom effects**	**Was Initial Control of this Venom Effect Achieved?**	**Vials of FabAV for Initial Control**
**Cohort Studies**				

**Prospective Data Collection**				

Bush 2002[10]	Patient 13^§^	Altered mental status	Yes	6
		Hypotension	Yes	
		Tachycardia	Yes	
		Neurotoxicity	Yes	
		SSS ≥ 7	Yes	
				
	Patient 14	Limb swelling	Yes	6
		Limb pain	Yes	
		Hypotension	Yes	
		Tachycardia	Yes	
		SSS ≥ 7	Yes	

Dart 1997[4]	Patient 10	SSS ≥ 7	Yes	8
		Platelet count < 50,000/mm^3^	Yes	
		Fibrinogen < 50 mg/dl*	Yes	

**Retrospective Data Collection**				

Lavonas 2004[25]	12-year-old Male	Compartment syndrome	Yes	4

Ruha 2002[13]	Patient 1	Limb swelling	Yes	18
		Limb pain	Yes	
		Protime > 50 sec	Yes	
		Fibrinogen < 50 mg/dl*	Yes	
		Platelet count < 50,000/mm^3^	Yes	

**Mixed Prospective + Retrospective Data Collection**				

Offerman 2002[20]	First coagulopathy patient^†^	Fibrinogen < 50 mg/dl*	Yes	NR
	Second coagulopathy patient^†^	Fibrinogen < 50 mg/dl*	Yes	NR
		Platelet count < 50,000/mm^3^	Yes	

**Non-Cohort Studies**				

Audi 2006[26]	80-year-old Female	Altered mental status	NR	6
		Hypotension	Yes	
		SSS ≥ 7	Yes	
		Platelet count < 50,000/mm^3^	Yes	

Camilleri 2005[27]	40-year-old Male	Limb swelling	Yes	N/A
		Limb pain	NR	
		Bleeding	No	

Clark 1997[22]	Case 1	Neurotoxicity	Yes	12
	Case 3	Neurotoxicity	Yes	6

Fazelat 2008[28]	24-year-old Male	SSS ≥ 7	Yes	10
		Platelet count < 50,000/mm^3^	Yes	
		Bleeding	No	

Gold 2004[29]	38-year-old Male	Platelet count < 50,000/mm^3^	No	N/A

Horn 2007[30]	8-year-old Male	Compartment syndrome	No	N/A

Lintner 2006[31]	2-year-old Male	Limb swelling	Yes	12
		Platelet count < 50,000/mm^3^	Yes	

Miller 2002[32]	Case 1	Limb swelling	NR	NR
		Platelet count < 50,000/mm^3^	Yes	

Morgan 2006[33]	14-year-old Male	Airway swelling	Yes	NR
		Altered mental status	Yes	
		Hypotension	Yes	
		Need for intubation	Yes	
		Bleeding	Yes	
		SSS ≥ 7	Yes	
		INR > 5.0	Yes	

Odeleye 2004[34]	Case 1	Platelet count < 50,000/mm^3^	No	N/A
	Case 2	Airway swelling	Yes	N/A
		Need for intubation	Yes	
		INR > 5.0	Yes	
		Platelet count < 50,000/mm^3^	No	

Offerman 2003[35]	42-year-old Male	Limb swelling	Yes	16
		Limb pain	Yes	
		Bleeding	NR	
		Platelet count < 50,000/mm^3^	Yes	

Richardson 2007[36]	Case 1	Need for intubation	No	N/A
		Neurotoxicity	No	
	Case 2	Neurotoxicity	No	N/A
		SSS ≥ 7	Yes	
		Platelet count < 50,000/mm^3^	Yes	

Seifert 1997[37]	53-year-old Female	Platelet count < 50,000/mm^3^	Yes	6

Wasserberger 2006[38]	46-year-old Male	Limb swelling	No	N/A
		Limb pain	No	
		Platelet count < 50,000/mm^3^	Yes	

^© ^This patient was also patient 6 in the Offerman, 2002 case series[20]

^† ^Drs. Bush and Offerman have confirmed that these patients and patients 13 and 14 in the Bush series are not the same subjects.

SSS = Snakebite Severity Score(Dart 96)

INR = International Normalized Ratio

NR = Not reported

N/A = Not applicable (initial control not obtained)

* Fibrinogen < 1.5 micromol/L

Five cohort studies and fourteen non-cohort studies were identified. Two of the cohort studies collected data prospectively, two collected data retrospectively, and one used both prospective and retrospective data collection. All of the non-cohort studies were of retrospective design. Seven severely envenomated patients were reported in the cohort studies, and 17 severely envenomated patients were described in the non-cohort studies.

### Initial response to FabAV therapy

Sixty-five specific severe venom effects were reported in these 24 patients. The initial response to FabAV treatment for these specific severe venom effects was: improved/resolved, 50 effects (77%); no improvement, 11 effects (17%); not reported, 4 effects (6%). All of the 22 specific venom effects (100%) experienced by the seven patients in the cohort studies improved after FabAV administration. In contrast, of the 43 specific venom effects experienced by the patients in the non-cohort studies, 28 effects improved or resolved (65%), 11 effects did not improve (26%), and the response of 4 effects were not reported (9%).

Initial control of the envenomation syndrome was achieved in 12 patients (50%), not achieved in 9 patients (38%), and not fully reported in 3 patients (13%). All seven (100%) of the severely envenomated patients in the cohort studies achieved initial control. Once again, the response to initial therapy was not as good in the patients reported in non-cohort studies. Among these 17 patients, initial control of the envenomation syndrome was achieved in 5 patients (29%), not achieved in 9 patients (53%), and incompletely documented in 3 patients (18%). The median dose of FabAV used to achieve initial control of the envenomation syndrome in these 12 patients was 6 vials (range: 4 – 18 vials).

### Persistent severe venom effects

One or more persistent severe venom effects were reported in 0/7 patients reported in cohort studies (0%), and in 9/17 patients in non-cohort reports (53%). These cases are summarized in Table 4[27-30,34,36,38]. These effects consisted of limb swelling, limb pain, soft tissue bleeding, thrombocytopenia, neurotoxicity, or compartment syndrome. Response to therapy was not reported for four patients, summarized in Table 5[26,27,32,35].

**Table 4:** Specific severe venom effects that persisted after FabAV administration

**Publication**	**Patient Identifying Information**	**Persistent Severe Venom Effects**	**Total Vials of FabAV**
**Cohort Studies (any design)**			

No cases			

**Non-Cohort Studies**			

Camilleri 2005[27]	40-year-old Male	Bleeding	32

Fazelat 2008[28]	24-year-old Male	Bleeding	26

Gold 2004[29]	38-year-old Male	Platelet count < 50,000/mm^3^	46

Horn 2007[30]	8-year-old Male	Compartment syndrome	10 (5 pre-operative)

Odeleye 2004[34]	Case 1	Platelet count < 50,000/mm^3^	22
	Case 2	Platelet count < 50,000/mm^3^	19

Richardson 2007[36]	Case 1	Need for intubation	22
		Neurotoxicity	
	Case 2	Neurotoxicity	10

Wasserberger 2006[38]	46-year-old Male	Limb swelling Limb pain	20

**Table 5:** Specific severe venom for which response to therapy was not documented

**Publication**	**Patient Identifying Information**	**Severe Venom Effects**	**Total Vials of FabAV**
**Cohort Studies (any design)**			

No cases			

**Non-Cohort Studies**			

Audi 2006[26]	80-year-old Female	Altered mental status	14

Camilleri 2005[27]	40-year-old Male	Limb pain	32

Miller 2002[32]	Case 1	Limb swelling	22

Offerman 2003[35]	42-year-old Male	Bleeding	48

### Recurrence or delayed onset of severe venom effects

Recurrence or delayed-onset of at least one severe venom effect was reported in 12 patients, including 3/7 patients (43%) in the cohort studies, described in Table 6[4,10,13,27,28,31,32,34-38]. Maintenance therapy was administered to two patients (17%), not administered to 9 patients (75%), and not documented for one patient (8%). With one exception (recurrent limb pain and swelling), all cases of recurrence or delayed onset of severe venom effects involved defibrination (with or without prothrombin time elevation) and/or thrombocytopenia, and were clinically occult. Although these events were judged *a priori *to represent "a severe threat of bleeding," none of the 11 patients (0%) with recurrent or delayed-onset hematologic venom effects developed bleeding.

**Table 6:** Recurrence or delayed onset of severe venom effects

**Publication**	**Patient Identifying Information**	**Vials of FabAV for Initial Control**	**Maintenance Therapy Administered?**	**Recurrent Severe Venom Effects**	**Delayed Onset Severe Venom Effects**
**Cohort Studies**					

**Prospective Data Collection**					

Bush 2002[10]	Patient 14	6	Yes	Limb swelling Limb pain	None

Dart 1997[4]	Patient 10	8	No	Fibrinogen < 50 mg/dl	None

**Retrospective Data Collection**					

Ruha 2002[13]	Patient 1	18	No	Protime > 50 sec	None
				Fibrinogen < 50 mg/dl	

**Mixed Prospective/Retrospective Data Collection**					

No cases					

**Non-Cohort Studies**					

Camilleri 2005[27]	40-year-old Male	N/A	No	None	Platelet count < 50,000/mm^3^ INR >5
					Fibrinogen < 50 mg/dl

Fazelat 2008[28]	24-year-old Male	10	Yes	Platelet count < 50,000/mm^3^	None

Lintner 2006[31]	2-year-old Male	12	No	Platelet count < 50,000/mm^3^	None

Miller 2002[32]	Case 1	NR	No	None	Fibrinogen < 50 mg/dl

Odeleye 2004[34]	Case 2	N/A	No	INR > 5.0	None

Offerman 2003[35]	42-year-old Male	16	NR	Platelet count < 50,000/mm^3^	None

Richardson 2007[36]	Case 1	N/A	No	None	Fibrinogen < 50 mg/dl

Seifert 1997[37]	53-year-old Female	6	No	Platelet count < 50,000/mm^3^	None

Wasserberger 2006[38]	46-year-old Male	N/A	No	Platelet count < 50,000/mm^3^	None

NR = Not reported

N/A = Not applicable (initial control not obtained)

### Permanent sequelae of envenomation

Few publications assessed and reported long-term outcomes. Therefore, the available data are inadequate to describe the long term outcomes after crotaline snakebite treated with FabAV. No published manuscripts described death following FabAV administration were identified in the literature search.

### Reports to the US National Poison Data System

The TESS/NPDS data include 21 deaths due to snakebite reported to participating US poison control centers from 2000 – 2006[1,14-19]. Of these, five patients received FabAV prior to death; two additional patients received unspecified antivenom. These cases are summarized in Table 7. Five patients presented *in extremis *and died of cerebral anoxia and/or multisystem organ failure; the other two patients died from complications of substance abuse.

**Table 7:** Reports of death after FabAV administration reported to the US National Poison Data System, 2000–2006

**Annual Report Year**	**Case Number**	**Clinical Summary**
**Patients Treated with FabAV**		

2001	55	A 17-year-old girl presented after apparent intravascular envenomation by an unknown snake with features of anaphylaxis to venom. Within minutes, she was hypotensive, profoundly hypoxic, developed pulmonary edema, and had a seizure. Treatment included intubation with ventilator support, dopamine, and FabAV (dose not reported). She never developed local tissue effects or coagulopathy. Despite maximal therapy, she died five days after envenomation.

2002	69	A 43-year-old man presented with severe hypotension after a rattlesnake bite. He remained hypotensive despite fluids, dopamine, epinephrine, diphenhydramine, steroids, and 26 vials of whole-IgG antivenom. Disseminated intravascular coagulopathy developed. He then received six vials of FabAV, after which his blood pressure improved such that vasopressors were discontinued. He developed epistaxis and oral mucosal bleeding that was treated with 7 additional vials of whole-IgG antivenom and transfusions of platelets, fresh frozen plasma, and cryoprecipitate (response not reported). He developed renal failure, and ultimately died of multi-system organ failure.

2003	424	A 33-year-old man received FabAV for an apparently uncomplicated rattlesnake envenomation. He was discharged from the hospital 24 hours later with only minimal symptoms, and was given a prescription for oxycodone with acetaminophen. He died of an apparent overdose of oxycodone that night.

2004	52	A 52-year-old man presented with a forearm compartment syndrome due to a copperhead snakebite. He received FabAV and underwent a dermotomy of his finger and a fasciotomy of his arm. Subsequently, he developed severe alcohol withdrawal, which was treated with diazepam, haloperidol, and fentanyl, which led to respiratory arrest, aspiration, and acute respiratory distress syndrome. Medical care was withdrawn, and the patient died.

2004	53	A 55-year-old man presented with severe hypotension and coma from apparent intravascular envenomation from a rattlesnake. FabAV therapy and aggressive resuscitation were not initiated for several hours. The patient developed renal failure, disseminated intravascular coagulopathy, and circulatory collapse, and died seven hours after envenomation.

**Patients Treated with Unspecified Antivenom**		

2001	56	A 30-year-old man presented comatose after rattlesnake envenomation. On presentation, he was found to have coagulopathy, thrombocytopenia, and a subarachnoid hemorrhage. He received "10 to 20 vials" of unspecified antivenom, but died after withdrawal of life support.

2005	57	A 32-year-old man presented to the emergency department in respiratory arrest due to anaphylaxis from a rattlesnake bite. He received unspecified antivenom, and subsequently died of anoxic encephalopathy.

Includes data from the Toxic Exposures Surveillance System (predecessor to the NPDS), 2000–2005.

## Discussion

Physicians in the United States treating victims bitten by rattlesnakes, cottonmouth and copperhead snakes, and pygmy rattlesnakes no longer have access to an antivenom that is licensed and approved to treat severely envenomated victims. The previous standard therapy, whole IgG antivenom, is no longer available; the currently-available antivenom, FabAV, was tested and approved only for use in mildly and moderately envenomated patients. Those patients with severe envenomation – hypotension, severe hematologic effects, and/or severe limb findings – are clinical "orphans." Data from the American Association of Poison Control Centers suggest that, when faced with the choice of off-label administration of FabAV or supportive care only, treating physicians most often choose to administer FabAV to severely envenomated patients[39]. It is difficult to conceive of a placebo-controlled trial of FabAV in severe snakebite; to our knowledge, no such study has been conducted.

A significant body of published literature describes the overall clinical experience with FabAV; this study identified 147 relevant articles, of which 19 articles provided case-level data about severely envenomated patients treated with FabAV.

Out of approximately ten published cohort studies of FabAV-treated patients, we were able to identify seven patients from five reports who met our *a priori *definition of severe envenomation. All seven of these patients demonstrated good initial response to FabAV therapy.

Severe snakebite is sometimes associated with the need for intubation, either due to airway edema or as part of supportive care of a patient in shock. Our review found three cases of severely envenomated patients who required intubation. One case involved a patient who was intubated for venom-induced periglottic edema, received FabAV, and was successfully extubated the next day[34]. A second case involved a patient who developed multisystem organ failure after deliberate intravenous injection of rattlesnake venom in a suicide attempt. The indication for his intubation, which occurred prior to FabAV therapy, was profound shock and gastrointestinal hemorrhage. These problems responded quickly to FabAV therapy; the patient successfully self-extubated on the third day of hospitalization[33]. We judged both these cases to demonstrate successful treatment of the venom effect, "need for intubation," with FabAV. The third case involved a patient who was intubated due to progressive neurotoxicity due to envenomation by an unknown rattlesnake. Administration of FabAV failed to prevent the need for intubation, and significant neurotoxicity progressed even after aggressive FabAV therapy[36].

Recurrence and delayed onset of severe venom effects are a known complication of snakebite, whether treated with FabAV or whole-IgG antivenom[5,40,41]. Cases involving both severe and initially-minor envenomation have been previously reported[41]. Three of the seven patients reported in the cohort studies developed recurrence phenomena. Two of these cases involved recurrent defibrination syndrome without bleeding; neither patient received maintenance FabAV therapy. Maintenance therapy has been shown in a randomized controlled trial to prevent early recurrence of local tissue venom effects. Use of maintenance therapy to prevent recurrent coagulopathy is based on strong pharmacokinetic arguments[5,12,37,42]. The third patient developed recurrent limb pain and swelling despite maintenance therapy.

Unfavourable outcomes, including severe venom effects that were refractory to the FabAV doses given, delayed-onset severe venom effects, and recurrence phenomena, were all reported more commonly in case reports and other non-cohort studies than in cohort studies. The difference was statistically significant (P = 0.005 for initial control, Fisher's Exact test). This is as expected; the presentation of novel results ("originality factor") increases the likelihood that a report will be accepted for presentation at a scientific meeting, and that it will subsequently be published as a full-length manuscript[43].

It is clear from these reports that, in some patients, defibrination and/or thrombocytopenia do not respond to large doses of FabAV. However, based on the observation that no reports describe medically significant bleeding that began after FabAV administration, the risk of bleeding in this situation is probably low. Subsequent to this structured literature review, a case report of fatal cerebral hemorrhage associated with recurrent defibrination in a FabAV-treated patient has been reported[44]. The authors of this report could find no other cases of significant spontaneous bleeding in 34 published cases of recurrent coagulopathy. A large cohort study of FabAV-treated patients, followed to resolution with detailed biochemical characterization including venom antigenemia, would be valuable to address this important question. Until that time, the recommendations made by Yip continue to represent the best available guidance in this area, with the *caveat *that, beyond a certain point, administration of additional FabAV to patients with refractory hemostatic dysfunction is unlikely to be beneficial[8].

The recommended dosing of FabAV is 4- to 6-vial aliquots, repeated as needed until the desired clinical effect is achieved. In the premarketing studies of mild and moderately envenomated patients, the median dose of FabAV used to achieve initial control of the envenomation syndrome was 6 vials (range: 3 to 12 vials)[4,5]. In this review of severely envenomated patients, initial control of severe venom effects was achieved after a median dose of 6 vials (range: 4 to 18 vials) was administered. Some patients received extraordinary doses in response to persistent or recurrent severe venom effects; whether patients benefited from doses in excess of 18 vials, excluding maintenance therapy, is unclear.

The NPDS contains information about 15,917 crotaline snake envenomations treated in a health care facility from 2000 to 2006; 21 of these patients (0.13%) died. Five fatality reports describe death that occurred after FabAV administration; another two patients received unspecified antivenom prior to death. No deaths appear to be caused by an adverse reaction to FabAV. Although it is difficult to make broad conclusions based on the sparse descriptions in these reports, the lack of any clear cases of treatment-failure associated death is reassuring. The fact that the fatal case reported by Kitchens and Eskin did not appear in the NPDS reports underscores one weakness of US poison-center based data, which rely on voluntary reporting[44].

In addition to issues surrounding retrospective data collection and publication bias, this report is limited by the lack of a comparison group. Published reports of severely envenomated North American crotalid victims managed without antivenom are extremely rare; therefore, it is difficult to differentiate the response to FabAV therapy from the natural history of untreated snakebite.

Because of the number of patients reported in cohort series is small, we cannot estimate the true rate of treatment failures, death, and other uncommon but important adverse events in the larger population of severely envenomated patients treated with FabAV.

No study reported in this series examined long-term outcomes.

One prior study, reported only in abstract form, has evaluated the use of FabAV in treatment of severe crotalid envenomation[45]. In that retrospective review of poison center cases, 9.3% of crotalid envenomations were judged to be severe. Initial control was achieved in 57% of severe cases, using a mean dose of 10.5 vials of FabAV.

As with any review, the conclusions of this report are limited by the available literature. To our knowledge, the largest cohort study of Crotaline snake bite victims treated with FabAV reported 93 cases[46]. A much larger, multi-center cohort study would be extremely useful to better define the answers to unresolved management issues.

## Conclusion

In this integrative review of the published literature, treatment of severely-envenomated crotalid snake victims with FabAV was generally associated with good short-term outcomes. Persistent, recurrent, or delayed-onset venom effects, particularly thrombocytopenia and defibrination, were observed in several patients, but no patient developed bleeding complications after receiving FabAV. FabAV therapy was well-tolerated. FabAV therapy appears to be appropriate in the management of severely envenomated crotalid snake victims.

## Abbreviations

FabAV: Crotaline Polyvalent Immune Fab (ovine); FDA: United States Food and Drug Administration; IgG: Immunoglobulin G; INR: International normalized ratio; N/A: Not applicable; NPDS: National Poison Data System; NR: Not Reported; SSS: Snakebite Severity Score; TESS: Toxic Exposures Surveillance System; US: United States (of America).

## Competing interests

This study was supported by a grant from Protherics, Inc, manufacturer of FabAV, to the Denver Health Hospital Authority. No author or other employee of the Denver Health Hospital Authority received direct or indirect compensation as a result of this grant or study.

## Authors' contributions

All authors participated in study design. SLM supervised the original library search. EJL reviewed these results and selected articles for hand review and data abstraction, which was performed by EJL and THS. SLM and JK managed the database. EJL wrote the manuscript draft. All authors read and approved the final manuscript.

## Pre-publication history

The pre-publication history for this paper can be accessed here:


